# Recent Progress on Hybrid Percolation Transitions

**DOI:** 10.3390/e28010068

**Published:** 2026-01-06

**Authors:** Young Sul Cho, Byungnam Kahng

**Affiliations:** 1 Department of Physics, Jeonbuk National University, Jeonju 54896, Republic of Korea; yscho@jbnu.ac.kr; 2 Center for Complex Systems, KI for Grid Modernization, Korea Institute of Energy Technology, Naju 58330, Republic of Korea

**Keywords:** hybrid percolation transition, hybrid phase transition, continuous percolation transition, explosive percolation transition, discontinuous percolation transition

## Abstract

Percolation describes the formation of a giant cluster once the average degree of a network exceeds a critical value. A hybrid percolation transition (HPT) denotes a phenomenon in which a discontinuous jump of the order parameter and the critical behavior, a basic pattern of a continuous transition, appear together at the same threshold. Such HPTs have been reported in many different systems. In this review, we present several representative examples of HPTs and classify them into two categories: global suppression-induced HPTs and cascading failure-induced HPTs. In the former class, critical behavior manifests itself in the distribution of cluster sizes, whereas in the latter it emerges in the distribution of avalanche sizes. We further outline the universal scaling relations shared by both types.

## 1. Introduction: Hybrid Percolation Transition

Percolation originally refers to the phenomenon in which a fluid penetrates a porous material and forms a path between its two opposite sides [1]. The percolation concept was applied to various natural and social phenomena, such as gelation in polymers [2,3,4], the spread of disease [5], the conductor-insulator transition [6,7], the resilience of systems [8,9], and the formation of public opinion [10,11]. To theoretically understand this phenomenon, one considers a network in which links are occupied according to a prescribed rule and analyzes the formation of clusters [12]. A percolation transition (PT) refers to the emergence of a giant cluster that contains a finite fraction of nodes O(N) with system size *N* when the mean degree *z* of a network exceeds a threshold $z_{c}$. The order parameter is m(z), defined as the fraction of nodes belonging to the largest cluster. The continuous PT (CPT) exhibits $m\left(z\right)∼{\left(z-z_{c}\right)}^{\beta }$ with criticality at $z_{c}$, where β is the critical exponent of the order parameter [13,14]. In discontinuous PT (DPT), m(z) jumps to $m_{0}$ at $z=z_{c}$ without exhibiting the critical behavior of a CPT.

Hybrid percolation transitions (HPT), sometimes referred to as mixed-order percolation transitions, describe the situation in which the features of CPT and DPT occur simultaneously at the same transition point $z_{c}$. Consequently, the order parameter m(z) behaves as

(1) $$m\left(z\right)=\left\{\begin{array}{cc} 0 & \mathrm{for}\;z<z_{c},\\ m_{0}+r{\left(z-z_{c}\right)}^{\beta } & \mathrm{for}\;z\geq z_{c}, \end{array}\right.$$

where $m_{0}$ and *r* are constants independent of *z*, and β is the critical exponent of the order parameter. Following the discontinuous jump of *m* at $z_{c}$, critical behavior analogous to that of a CPT appears and is described by

(2) $$n_{s}=s^{-\tau }f\left[s{\left(z-z_{c}\right)}^{1/\sigma }\right]\;\;\;\mathrm{for}\;z\geq z_{c},$$

where $n_{s}$ is the distribution of finite clusters of size *s* or finite avalanches of size *s*, and τ and σ are critical exponents associated with the size distribution [15,16].

In this review, we highlight two types of HPTs: global-suppression-induced HPTs and cascading-failure-induced HPTs. For the first type of HPTs, critical behavior is observed in the cluster-size distribution, as discussed in Section 2. For the second type, critical behavior appears in the avalanche size distribution in interdependent networks and in *k*-core percolation, as discussed in Section 3.

## 2. Global-Suppression-Induced Hybrid Percolation Transition

In a random network or lattice, links are added one at a time, and a cluster is defined as a group of nodes that can reach each other via these links. In explosive percolation (EP) models, the formation of large clusters is hindered by choosing, at each step, an optimal link from a set of candidate links. When the number of candidate links is finite [17,18,19,20,21,22,23,24,25,26], the model shows a CPT. In contrast, for an infinite number of candidate links, it exhibits either a DPT or a hybrid percolation transition (HPT) [27,28,29,30,31,32,33]. In this work, suppression based on an infinite pool of candidate links is referred to as global suppression.

We summarize two models, the half-restricted model (HR) [29] and the Bohman-Frieze-Wormald model (BFW) [34], which exhibit an HPT driven by global suppression. The criticality observed at $z_{c}$ arises from a tug-of-war type of competition between two sets for $z<z_{c}$ in the first model [35], and from the duration for which the cluster of a given node persists before merging with another cluster in the second model [36].

### 2.1. Half-Restricted Model

**Figure 1:**
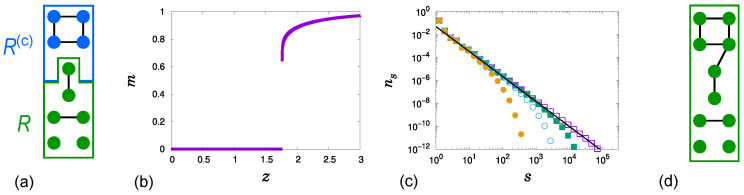
(**a**) An illustration of the division into two subsets, *R* (the lower 6 nodes marked in green) and $R^{\left(c\right)}$ (the upper 4 nodes marked in blue), is provided for N=10 and g=0.5. Note that all 6 nodes are assigned to *R* even though gN=5, since *R* is defined to contain every node in the marginal cluster. (**b**) Plot of *m* for g=0.5. (**c**) Plot of $n_{s}$ for g=0.5 at $z=z_{c}$ (□), and for successively larger values of $z>z_{c}$, shown from right to left. The solid line has slope −2.18. (**d**) In this case, all 10 nodes are included in *R* because the largest cluster exceeds gN=5 in size.

The original HR model was proposed in [29] to generate a DPT in an EP model with global suppression. In this study, we investigate a modified HR model constructed to make the HPT more distinctly visible, following the analytical considerations of [33]. We consider a network composed of *N* nodes, controlled by an external parameter 0<g≤1. The network is initially empty (no links), and at each time step exactly one link is added, so that a total of zN/2 links are eventually introduced. To establish each link, we select one node uniformly at random from the entire set of nodes, and the other node from a restricted subset *R*, defined as follows. All clusters $\left\{c_{\ell }\;|\;\ell \geq 1\right\}$ in the network are ordered by nondecreasing size, so that $s_{\ell }\leq s_{\ell +1}$ for all ℓ≥1, where $s_{\ell }$ denotes the size of $c_{\ell }$. The restricted node set *R* consists of all nodes that belong to the smallest clusters $\left\{c_{1},c_{2},\ldots ,c_{k}\right\}$, where *k* is chosen such that $\sum_{\ell =1}^{k-1}s_{\ell }<\left\lfloor gN\right\rfloor \leq \sum_{\ell =1}^{k}s_{\ell }$, and ⌊gN⌋ is the greatest integer not exceeding gN. In other words, *R* contains approximately a fraction *g* of the nodes, all of which are in the smallest clusters. In this modified version, *R* includes every node in the marginal cluster $c_{k}$, whereas in the original HR model only a subset of nodes from $c_{k}$ is taken so that the total size of *R* is exactly ⌊gN⌋. A schematic illustration of the division into the two sets *R* and $R^{\left(c\right)}$ is provided in Figure 1a.

When g<1, *m* exhibits an HPT of the form given in Equation (1), as shown in Figure 1b. The cluster size distribution $n_{s}$ also exhibits a critical behavior Equation (2) as $z\to z_{c}^{+}$, as shown in Figure 1c. In [33], the scaling relations β=τ−2 and γ=3−τ with σ=1 were derived independently of *g*, where γ characterizes the critical behavior of the mean size of finite clusters, $\sum_{s}s^{2}n_{s}\left(z\right)∼{\left(z-z_{c}\right)}^{-\gamma }$ as $z\to z_{c}^{+}$. Moreover, τ continuously increases from 2 to 2.5 as *g* increases from 0 to 1. Therefore, the critical exponents β and γ vary continuously for a fixed σ=1, following β=τ−2 and γ=3−τ as *g* increases. The rate equation for $n_{s}$ for $z<z_{c}$, along with a qualitative explanation of how τ varies as *g* increases based on this rate equation, is presented in [33].

The derivation of the relations β=τ−2,γ=3−τ, and σ=1 is summarized as follows. At $z=z_{c}$, numerical results show that $m\left(z_{c}\right)=m_{0}>1-g$, implying that the largest cluster is $c_{k}$ and its size exceeds (1−g)N. Consequently, *R* contains all nodes, as illustrated in Figure 1d. Therefore, for $z\geq z_{c}$, the HR model follows ER-type link addition, in which links are added between two randomly selected nodes.

The rate equation for ER-type link addition, written in terms of the shifted time $\hat{z}\equiv z-z_{c}$, is

(3) $$\frac{dn_{s}\left(\hat{z}\right)}{d\hat{z}}=\frac{1}{2}\sum_{i+j=s}ijn_{i}\left(\hat{z}\right)n_{j}\left(\hat{z}\right)-sn_{s}\left(\hat{z}\right)\;\;\mathrm{for}\;\hat{z}\geq 0.$$

The initial condition is $n_{s}\left(\hat{z}=0\right)=a_{0}s^{-\tau }$, where $a_{0}=\left(1-m_{0}\right)/\zeta \left(\tau -1\right)$ follows from $\sum_{s=1}sn_{s}\left(\hat{z}=0\right)=1-m_{0}$. Using the generating function $g_{1}\left(\mu ,\hat{z}\right)=\sum_{s=1}^{\infty }sn_{s}\left(\hat{z}\right)e^{\mu s}$, Equation (3) can be written as ${\dot{g}}_{1}\left(\mu ,\hat{z}\right)=2g_{1}^{\prime }\left(\mu ,\hat{z}\right)\left(g_{1}\left(\mu ,\hat{z}\right)-1\right)$, where ${\dot{g}}_{1}\left(\mu ,\hat{z}\right)$ and $g_{1}^{\prime }\left(\mu ,\hat{z}\right)$ denote derivatives of $g_{1}\left(\mu ,\hat{z}\right)$ with respect to $\hat{z}$ and μ, respectively. The solution of this equation is given by

(4) $$g_{1}\left(\mu ,\hat{z}\right)=1-H\left(-\mu -\hat{z}\left(g_{1}\left(\mu ,\hat{z}\right)-1\right)\right)$$

referring to the F-model in [37], where $H\left(x\right)=1-\sum_{s=1}sn_{s}\left(\hat{z}=0\right)e^{-xs}=1-a_{0}\sum_{s=1}s^{1-\tau }e^{-xs}$.

The order parameter is $m\left(\hat{z}\right)=1-g_{1}\left(0^{-},\hat{z}\right)$, since $g_{1}\left(0^{-},\hat{z}\right)=\sum_{s=1}sn_{s}\left(\hat{z}\right)$ is the fraction of nodes belonging to finite clusters. By inserting $\mu =0^{-}$ into Equation (4), one obtains the self-consistency equation

(5) $$m\left(\hat{z}\right)=H\left(\hat{z}m\left(\hat{z}\right)\right).$$

To obtain the critical behavior as $\hat{z}\to 0^{+}$, $H(\hat{z}m)$ is expanded for small $\hat{z}m$ as $H\left(\hat{z}m\right)=1-a_{0}\left[\zeta \left(\tau -1\right)+\Gamma \left(2-\tau \right){\left(\hat{z}m\right)}^{\tau -2}\right]$. Substituting this expansion into the right-hand side of Equation (5) yields

(6) $$m-m_{0}∼{\hat{z}}^{\tau -2},$$

using the condition $m\left(\hat{z}=0\right)=m_{0}>0$. Comparing with the critical behavior $m-m_{0}∼{\hat{z}}^{\beta }$, the relation β=τ−2 is obtained.

To obtain the relation γ=3−τ, we use the identity $g_{1}^{\prime }\left(0^{-},\hat{z}\right)=\sum_{s}s^{2}n_{s}\left(\hat{z}\right)$. From Equation (4), we obtain $g_{1}^{\prime }\left(0^{-},\hat{z}\right)=H^{\prime }\left(\hat{z}m\right)\left[1+\hat{z}g_{1}^{\prime }\left(0^{-},\hat{z}\right)\right]$, which leads to

(7) $$g_{1}^{\prime }\left(0^{-},\hat{z}\right)=\frac{H^{\prime }\left(\hat{z}m\left(\hat{z}\right)\right)}{1-\hat{z}H^{\prime }\left(\hat{z}m\left(\hat{z}\right)\right)},$$

where $H^{\prime }\left(x\right)$ denotes the derivative of *H* with respect to *x*.

$H^{\prime }\left(x\right)=a_{0}\sum_{s=1}s^{2-\tau }e^{-xs}$ is expanded for small $x=\hat{z}m$. Keeping only the leading contribution, we obtain $H^{\prime }\left(\hat{z}m\right)=a_{0}\Gamma \left(3-\tau \right){\left(\hat{z}m\right)}^{\tau -3}$, since 2<τ<2.5. By inserting the leading contribution to the right-hand side of Equation (7), we obtain

(8) $$\sum_{s=1}s^{2}n_{s}∼{\hat{z}}^{-(3-\tau )},$$

using the condition $m\left(\hat{z}=0\right)=m_{0}>0$. This yields γ=3−τ by comparison with the critical behavior $\sum_{s=1}s^{2}n_{s}∼{\left(z-z_{c}\right)}^{-\gamma }$. We note that $\hat{z}H^{\prime }\left(\hat{z}m\right)$ in the denominator vanishes as $\hat{z}\to 0^{+}$ since 2<τ<2.5.

σ=1 in Equation (2) is derived by expanding $n_{s}\left(\hat{z}\right)=s^{-\tau }\sum_{\ell =0}a_{\ell }{\left(s^{\sigma }\hat{z}\right)}^{\ell }$, where $a_{\ell }$ are $\hat{z}$-independent constants. By inserting this expansion into Equation (3) and comparing terms of the same order in $\hat{z}$ on both sides, together with the constraint $1-a_{0}\sum_{s=1}s^{1-\tau }=m_{0}>0$, one obtains σ=1. Thus, $n_{s}=s^{-\tau }\sum_{\ell =0}a_{\ell }{\left(s\hat{z}\right)}^{\ell }=s^{-\tau }f\left(s\hat{z}\right)$ satisfies Equation (2) with σ=1. We note that σ=1 also confirms the conventional scaling relations β=(τ−2)/σ and γ=(3−τ)/σ.

The HR model evolves through a forward link-addition process in which *z* increases irreversibly. Consequently, the exponent τ must be governed by the properties of the regime $z<z_{c}$. In a subsequent study [35], this mechanism was clarified by analyzing the interevent time distribution $P_{d}\left(z\right)$, defined as the distribution of the durations *z* for which each node stays in the set *R* or $R^{\left(c\right)}$ before transitioning to the other set. As *m* undergoes a sudden rise up to $m=g<m_{0}$ near $z_{c}$, $P_{d}\left(z\right)$ displays a power-law tail, $P_{d}\left(z\right)∼z^{-\zeta^{\prime }}$.

To obtain an expression for $\zeta^{\prime }$, the mean waiting time *z* of a cluster of size *s* in set *R* is calculated as z=g/[(1+g)s]. During the short time interval near $z_{c}$, a cluster of size *s* is selected with a probability proportional to $sn_{s}\left(z_{c}\right)\propto s^{1-\tau }$. A nodewise measurement for $P_{d}\left(z\right)$ introduces an additional factor of *s*. In conclusion,

(9) $$P_{d}\left(z\right)=s\cdot sn_{s}\left|\frac{\partial s}{\partial z}\right|∼s^{-(\tau -4)}∼z^{-(4-\tau )},$$

which yields $\zeta^{\prime }=4-\tau$. This indicates that the critical behavior at $z_{c}$ is governed by the cluster coalescence dynamics occurring for $z<z_{c}$. Detailed derivations are presented in [35].

In a subsequent study [36], two distinct sets of critical exponents for $z\to z_{c}^{+}$, namely $\left\{\beta_{m},\gamma_{m},{\bar{\nu }}_{m}\right\}$ and $\left\{\tau_{s},\sigma_{s},\gamma_{s},{\bar{\nu }}_{s}\right\}$, were proposed to describe, respectively, the critical behavior of the order parameter and that of the cluster size distribution. The first set is associated with the scaling relations $m-m_{0}∼{\left(z-z_{c}\right)}^{\beta_{m}}$, $N\left(\langle m^{2}\rangle -{\langle m\rangle }^{2}\right)∼{\left(z-z_{c}\right)}^{-\gamma_{m}}$, and $m\left(z_{c}\left(N\right)\right)-m_{0}∼N^{-\beta_{m}/{\bar{\nu }}_{m}}$, where 〈·〉 denotes an ensemble average and $z_{c}\left(N\right)-z_{c}∼N^{-1/{\bar{\nu }}_{m}}$. For the second set, $\tau_{s}$ and $\sigma_{s}$ play the roles of the critical exponents in Equation (2) via the identifications $\tau \equiv \tau_{s}$ and $\sigma \equiv \sigma_{s}$. The characteristic cluster size follows $s^{*}∼{\left(z-z_{c}\right)}^{-1/\sigma_{s}}$, and at $z_{c}\left(N\right)$ it scales as $s^{*}\left(z_{c}\left(N\right)\right)∼N^{1/\sigma_{s}{\bar{\nu }}_{s}}$ when $z_{c}\left(N\right)-z_{c}∼N^{-1/{\bar{\nu }}_{s}}$. The exponent $\gamma_{s}$ governs the critical behavior of the mean size of finite clusters, given by $\sum_{s}s^{2}n_{s}\left(z\right)∼{\left(z-z_{c}\right)}^{-\gamma_{s}}$.

The two sets of critical exponents resulted in the derivation of three scaling relations: ${\bar{\nu }}_{m}=2\beta_{m}+\gamma_{m}$ within the first set, $\gamma_{s}=\left(3-\tau_{s}\right)/\sigma_{s}$ within the second set, and $\gamma_{s}+\beta_{m}=1$ between the two sets, where $\sigma \equiv \sigma_{s}=1$ as discussed above.The first two scaling relations are explained by the conventional scaling relations $\bar{\nu }=2\beta +\gamma$ and γ=(3−τ)/σ within each set. The third relation $\gamma_{s}+\beta_{m}=1$ is given by

(10) $$\frac{dm}{dz}=\sum_{s}s^{2}n_{s}$$

which expresses that the increment of the order parameter equals the average size of the finite cluster [36]. In Table 1, the critical exponent values of the HR model for various values of *g* are listed. These results confirm the three scaling relations, as well as the relation $\tau_{s}=4-\zeta^{\prime }$ introduced above, with the identification $\tau \equiv \tau_{s}$.

### 2.2. BFW Model

The original BFW model was proposed in [34] to introduce a DPT in an EP model with global suppression. Here, we consider a modified version of BFW model to enable a clearer observation of the HPT based on analytical results [36]. In a network of *N* nodes, an external parameter 0<h≤1 is determined. Initially, the network contains no links, and the parameters are set to k=2 and u=0. Links are added according to the following rules and the number of added links is L=zN/2.

At each time step u→u+1, two nodes from different clusters are randomly selected, and the sizes of their respective clusters are denoted by $s_{1}$ and $s_{2}$. A link between the two nodes is then either added or rejected according to the following rules:

(i) If $s_{1}+s_{2}\leq k$ or $s_{1}+s_{2}>k$ but L/u≤h, a link is added and $k\to s_{1}+s_{2}$.
(ii) Otherwise, a link is not added.

Therefore, both *k* and *u* evolve as *u* increases. We note that in the original BFW model, *h* depends on *k* as $h\left(k\right)=1/2+{\left(2k\right)}^{-1/2}$, whereas in the present model *h* remains constant throughout the process.

When h<1, *m* exibits a HPT of the form given in Equation (1). The BFW model shows the same qualitative features of an HPT as the HR model, but with different exponent values. Two sets of critical exponents $\left\{\beta_{m},\gamma_{m},{\bar{\nu }}_{m}\right\}$ and $\left\{\tau_{s},\sigma_{s},\gamma_{s},{\bar{\nu }}_{s}\right\}$ were identified along with the three scaling relations ${\bar{\nu }}_{m}=2\beta_{m}+\gamma_{m}$, $\gamma_{s}=\left(3-\tau_{s}\right)/\sigma_{s}$, and $\gamma_{s}+\beta_{m}=1$. Here, the relations $\beta_{m}=\tau_{s}-2,\gamma_{s}=3-\tau_{s}$, and $\sigma_{s}=1$ were derived, independent of the value of *h*. To obtain these results, one uses the fact that the probability of link rejection in (ii) becomes zero for $z\geq z_{c}$, so that ER-type link addition applies with the initial conditions $n_{s}\left(z_{c}\right)∼s^{-\tau_{s}}$ and $m\left(z_{c}\right)=m_{0}>0$. The detailed derivation can be followed from Equations (3)–(10) by identifying $\tau \equiv \tau_{s}$, $\sigma \equiv \sigma_{s}$, $\beta \equiv \beta_{m}$, and $\gamma \equiv \gamma_{s}$. Like the HR model, τ increases continuously from 2 to 2.5 as *h* increases from 0 to 1. Consequently, for the fixed value σ=1, the exponents β and γ also vary continuously as *h* increases.

To explain the exponent $\tau_{s}$ at $z=z_{c}$ in terms of the dynamics for $z<z_{c}$, the age distribution $P_{d}\left(z\right)$ was analyzed. Here, the age *z* is defined as the time interval during which the cluster of a given node is not connected to any other cluster. During the abrupt increase of *m* up to $m(z_{b})$ on the verge of $z_{c}$, $P_{d}\left(z\right)$ exhibits a power-law decay $P_{d}\left(z\right)∼z^{-\zeta^{\prime }}$, where $z_{b}$ is the onset of the ER-type link addition. It was shown that $\tau =4-\zeta^{\prime }$ which implies that the critical behavior at $z_{c}$ is indeed characterized by the cluster coalescence before $z_{c}$. $P_{d}\left(z\right)$ follows dynamics similar to those of the HR model, and thus the origin of the relation $\tau =4-\zeta^{\prime }$ can be understood using Equation (9).

In Table 2, the critical exponent values of the BFW model for various values of *h* are listed. These results confirm the scaling relations mentioned above.

## 3. Cascading-Failure-Induced Hybrid Percolation Transition

### 3.1. Percolation in Interdependent Networks

In [38], the authors investigated percolation in a pair of interdependent networks, each containing the same number of nodes *N* and sharing the same average degree *z*. Within each network, nodes are linked by connectivity links, and every node in one network is connected to a corresponding node in the other network via a dependency link. When *z* exceeds a critical threshold, the mutually giant connected component (MGCC) appears in a discontinuous manner. Here, a mutually connected component (MCC) is defined as a collection of dependent node pairs such that, within each individual network, all nodes in the component are reachable from one another through connectivity links. The order parameter m(z) is given by the fraction of nodes that are part of the largest MCC.

Percolation phenomena in interdependent networks have been observed in a range of systems, including ferromagnetic and superconducting networks [39,40]. While subsequent studies have extended the framework to more than two interdependent networks [41,42,43], in this work we restrict our analysis to the case of two interdependent networks.

We focus on a scenario in which each pair of mutually dependent nodes is chosen at random. On a variety of network structures, the MGCC vanishes discontinuously as *z* is reduced below $z_{c}$ in accordance with Equation (1). Because this discontinuous jump in the order parameter is accompanied by critical behavior characteristic of a continuous transition, the transition is classified as hybrid, as elaborated below.

**Figure 2:**
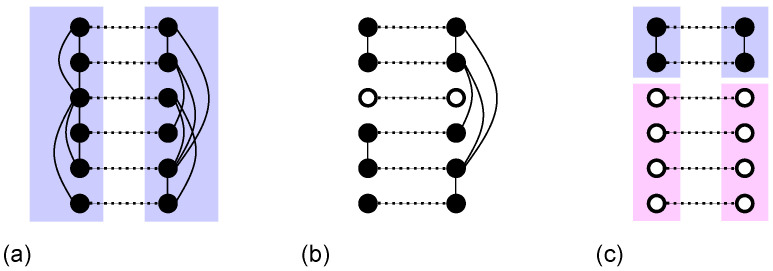
Example of cascade size in the percolation of interdependent networks. Solid lines represent connectivity links, and dotted lines represent dependency links. (**a**) Initially, all nodes in each network belong to a giant mutually connected component. (**b**) A pair of interdependent nodes (∘) is removed. (**c**) The two upper nodes in each network remain in a mutually connected component. The other four nodes determine the avalanche size.

Consider a pair of interdependent networks with $z>z_{c}$ that has a MGCC. We first remove a randomly chosen pair of nodes from this MGCC. Subsequently, a cascade of failures of dependent node pairs typically unfolds: whenever one node in a pair loses its connection to the MGCC in its own network, the entire pair fails. This process proceeds iteratively until each dependent pair is either fully included in the MGCC or fully excluded from it. The size of the avalanche is defined as the total number of nodes that are disconnected from the MGCC during this entire cascade. An illustrative avalanche event is presented in Figure 2.

The avalanche size distribution $n_{s}$ represents the probability that an avalanche triggered by the removal of a pair of nodes has size *s*. To obtain $n_{s}$, one removes different node pairs from given interdependent networks and then averages the resulting avalanche sizes over many network realizations. For a variety of networks, this avalanche size distribution shows the critical behavior given in Equation (2) as $z\to z_{c}^{+}$ [15,44].

The critical properties described in Equations (1) and (2) have been investigated for a variety of interdependent network structures, including interdependent scale-free (SF), Erdős–Rényi (ER), and two-dimensional square lattice (2D) networks. For interdependent SF and ER networks, the systems can be approximated as locally tree-like, leading to the exponents β=1/2 and τ=3/2 [15]. Notably, a HPT occurs even in SF networks with a degree exponent smaller than 3, in contrast to ordinary percolation on a single SF network, where the giant component appears continuously starting from $z_{c}=0$. For interdependent 2D networks, numerical simulations yield β=0.53±0.02 and τ=1.59±0.02 [44].

In a subsequent investigation of the critical behavior [44], two sets of critical exponents, $\left\{\beta_{m},\gamma_{m},{\bar{\nu }}_{m}\right\}$ and $\left\{\tau_{a},\sigma_{a},\gamma_{a},{\bar{\nu }}_{a}\right\}$, were introduced to characterize the behavior as $z\to z_{c}^{+}$. The first set is associated with the order parameter and satisfies the scaling relations $m-m_{0}∼{\left(z-z_{c}\right)}^{\beta_{m}}$, $N\left(\langle m^{2}\rangle -{\langle m\rangle }^{2}\right)∼{\left(z-z_{c}\right)}^{-\gamma_{m}}$, and $m\left(z_{c}\left(N\right)\right)-m_{0}∼N^{-\beta_{m}/{\bar{\nu }}_{m}}$, where 〈·〉 denotes an ensemble average and $z_{c}\left(N\right)-z_{c}∼N^{-1/{\bar{\nu }}_{m}}$.

For the second set, $\tau_{a}$ and $\sigma_{a}$ are identified with the exponents appearing in Equation (2) by setting $\tau \equiv \tau_{a}$ and $\sigma \equiv \sigma_{a}$. The characteristic cluster size scales as $s^{*}∼{\left(z-z_{c}\right)}^{-1/\sigma_{a}}$, and at the finite-size threshold $z_{c}\left(N\right)$ it follows $s^{*}\left(z_{c}\left(N\right)\right)∼N^{1/\sigma_{s}{\bar{\nu }}_{a}}$, where $z_{c}\left(N\right)-z_{c}∼N^{-1/{\bar{\nu }}_{a}}$. The exponent $\gamma_{a}$ describes the critical scaling of the mean size of finite avalanches, $\sum_{s}sn_{s}\left(z\right)∼{\left(z-z_{c}\right)}^{-\gamma_{a}}$.

These two exponent sets are not independent; they are connected through the relation $m\left(z\right)+\int_{z}^{z_{0}}\left[\sum_{s}sn_{s}\left(\tilde{z}\right)\right]d\tilde{z}=m\left(z_{0}\right)$, where $z_{0}$ denotes the value of *z* at the onset of the cascading failure. Differentiating this relation yields $dm/dz=\sum_{s}sn_{s}\left(z\right)$ and leads to

(11) $$1-\beta_{m}=\gamma_{a}.$$

Table 3 summarizes the numerically evaluated critical exponents for interdependent ER and 2D networks. Notably, the hyperscaling relation ${\bar{\nu }}_{m}=2\beta_{m}+\gamma_{m}$ is satisfied within the quoted uncertainties. In addition, the relation given in Equation (11) also holds within the error ranges, as expected.

### 3.2. *k*-Core Percolation

A *k*-core is a subgraph in which every node has at least *k* neighbors that also lie within this subgraph. When links are added among *N* nodes and the average degree *z* surpasses a critical value $z_{c}$, a giant *k*-core appears. *k*-core percolation (also known as bootstrap percolation) was first examined on the Bethe lattice [45]. Later studies extended this analysis to locally tree-like networks, including ER and SF networks [46,47,48]. The order parameter m(z) is defined as the fraction of nodes that are part of the largest *k*-core.

For k≥3, the giant *k*-core disappears discontinuously as *z* decreases below $z_{c}$ following Equation (1) on the Bethe lattice and ER network. This discontinuity is accompanied by critical behavior, indicating that the transition is hybrid. We note that *k*-core percolation exhibits a continuous transition in SF networks with a degree exponent below 3.

At t=0, a network with $z>z_{c}$ is given that contains a giant *k*-core. At each time step t→t+1, all nodes with degrees less than *k* are removed. Removing a node reduces the degrees of its neighbors, and some of those neighbors may then have degrees less than *k*, which causes them to be removed in the next step. This cascading process continues until only the *k*-cores remain.

**Figure 3:**
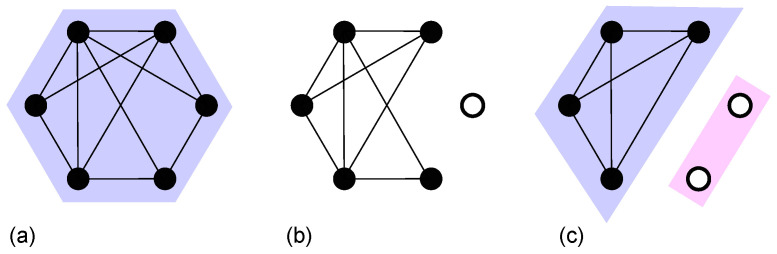
Example of cascading failures in *k*-core percolation for k=3. (**a**) Initially, all nodes belong to a giant *k*-core. (**b**) One node (∘) is removed. (**c**) Four nodes form a *k*-core, and the other two isolated nodes determine the avalanche size.

The critical behavior of the *k*-core pruning process has been understood as a branching process with a mean branching coefficient close to 1 in uncorrelated, locally tree-like networks [47]. In the ER network, the dynamics of the pruning process have been studied in three regimes: $z<z_{c}$, $z>z_{c}$, and $z=z_{c}$. For $z<z_{c}\left(z>z_{c}\right)$ the duration (relaxation) time until the steady state diverges as $|z-z_{c}{|}^{-1/2}$. At $z=z_{c}$, the probability that a vertex is removed at time *t* decays as $t^{-2}$. The avalanche size distribution $n_{s}$ triggered by the removal of a node in the giant *k*-core (See Figure 3 for an example.) also follows Equation (2) with τ=3/2 as $z\to z_{c}^{+}$.

In a subsequent investigation of critical behavior [48], two sets of critical exponents, $\left\{\beta_{m},\gamma_{m},{\bar{\nu }}_{m}\right\}$ and $\left\{\tau_{a},\sigma_{a},\gamma_{a},{\bar{\nu }}_{a}\right\}$, which were originally introduced for interdependent-network percolation, were analyzed in the context of *k*-core percolation. It was found that characteristic features, including the scaling relations in Equation (11) and ${\bar{\nu }}_{m}=2\beta_{m}+\gamma_{m}$, also hold for *k*-core percolation, as verified by measurements on ER networks summarized in Table 4. These universal aspects, shared by interdependent-network percolation and *k*-core percolation, are accounted for within the unified framework described in Section 3.3.

### 3.3. Unified Framework as a Critical Branching Process of the Two-Step Contagion Model

In [16], the two-step contagion model was adopted to explain the underlying dynamics of the hybrid percolation transitions in interdependent networks and *k*-core percolation within a unified framework. In the two-step contagion model, each node can be in one of four states: susceptible (*S*), weakened (*W*), infected (*I*), or removed (*R*). Consequently, the model is known as the SWIR model [49].

**Figure 4:**
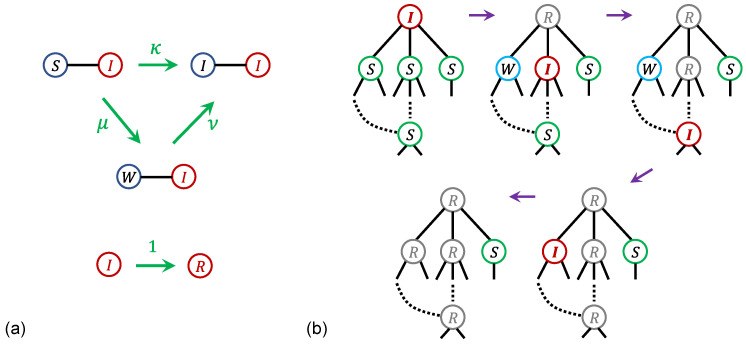
(**a**) Schematic diagram of the update rules for nodes in the SWIR model. (**b**) Schematic diagram of the spread of infection originating from the top node and following the arrows. During the third step, the *W* node is infected via the long range shortcut.

The dynamical rules of the model are defined as follows: Consider a network with a mean degree *z*. At time t=0, one node is initialized in state *I*, while all remaining nodes are in state *S*. At each subsequent time *t*, the following procedure is applied to every node that is in state *I* at that time. For each such infected node, the states of all of its neighbors are updated for the next time step according to these rules. If a neighbor is in state *S*, it changes to state *I* with probability κ or to state *W* with probability μ. If a neighbor is in state *W*, it transitions to state *I* with probability ν. These neighbor update rules are depicted in Figure 4a. To encode that weakened nodes are more susceptible to infection, the model imposes ν>κ. As the system progresses from *t* to t+1, all nodes that were in state *I* at time *t* transition to state *R*. This update procedure for each time step t→t+1 is iterated until the system reaches an absorbent configuration in which no infected nodes remain in the network.

For a fixed value of κ, the infection of *S* nodes propagates outward from the initially infected node as time *t* increases. If $\kappa <\kappa_{c}$, the total number of removed nodes in the absorbing state stays finite, corresponding to a finite avalanche. In contrast, when $\kappa >\kappa_{c}$, the total number of removed nodes in the absorbing state scales as O(N), indicating an infinite avalanche. Notably, a sharp rise in the number of removed nodes appears around $t\propto N^{1/3}$, because $O(N^{2/3})$ of the *W* nodes become infected via long-range shortcuts, as depicted in Figure 4b. The order parameter m(κ) is defined as the fraction of *R* nodes in the absorbing state. For $z>2/(\sqrt{\mu^{2}+4\mu \nu }-\mu )$, m(κ) displays a hybrid phase transition, following $m-m_{0}∼{\left(\kappa -\kappa_{c}\right)}^{\beta }$ for $\kappa >\kappa_{c}$.

At $\kappa =\kappa_{c}$, the infection originating from a single initially infected node evolves as a critical branching process, with an average branching factor close to 1. This behavior is a typical feature in both interdependent network percolation and *k*-core percolation, offering a common explanation for the fundamental mechanism driving criticality in these two models.

In the percolation of interdependent networks, the unbounded cascade of link failures at $z=z_{c}$, initiated by removing a node from the MGCC, can be viewed as the critical branching process of the SWIR model. The connectivity links in the two interdependent networks are denoted as *A*-type and *B*-type links. For comparison with the SWIR model on a single network, we treat each pair of mutually dependent nodes as one effective node carrying both *A*-type and *B*-type links. The corresponding effective degrees $k_{A}\left(j\right)$ and $k_{B}\left(j\right)$ are then assigned to each node *j* for the respective link types, such that every node in the MGCC satisfies $k_{A}\geq 1$ and $k_{B}\geq 1$.

At each time *t*, each node is considered to be in one of the four states of the SWIR model. When a node is removed, it can trigger the failure of links connected to its neighbors; therefore, it is treated as a *I*-state node at that time and transitions to a *R*-state node at the next step. Initially, at t=0, every node with $k_{A}=1$ or $k_{B}=1$ is placed in the *S*-state, since it can be removed at the following time step if one of its neighboring nodes is in the *I* state, and therefore it becomes an *I*-state node at the next time. During the avalanche (t≥1), any node that newly acquires $k_{A}=1$ or $k_{B}=1$ is classified as a *W*-state node. With this mapping, it has been verified in ER networks that the dynamics of the avalanche at $z=z_{c}$ corresponds to the critical branching process of the SWIR model, characterized by a mean branching coefficient close to 1.

In *k*-core percolation, the unbounded cascade of node failures occurring at $z=z_{c}$ after the removal of a node in the giant *k*-core can be understood as the critical branching process of the SWIR model. Analogous to the framework used for interdependent networks, each node in *k*-core percolation is assumed to occupy one of the four SWIR states at each time step *t*. When a node is removed, it may cause the links of its neighboring nodes to fail; hence it is treated as an *I*-state node and then transitions to an *R*-state node in the next step. At the initial time t=0, every node *i* with degree $k_{i}=k$ is placed in the *S*-state, because it can be removed at the next step as a consequence of the removal of one of its *I*-state neighbors, thereby becoming an *I*-state node in the following step. During the avalanche (t≥1), if a node *i* attains degree $k_{i}=k$, it is classified as a *W*-state node. Within this mapping, it has been verified for ER networks with k=3 that the avalanche dynamics at $z=z_{c}$ are captured by the critical branching process of the SWIR model, characterized by a mean branching coefficient close to 1.

## 4. Summary

In this review, we have summarized recent advances on two classes of HPTs in Section 2 and Section 3. The first class involves an HPT of a giant cluster that forms under global suppression as links are added. In this scenario, the critical behavior at $z_{c}$ is captured by the scaling of the cluster size distribution. Because both the HR and BFW models evolve as forward processes, their critical exponents at $z_{c}$ are determined, for $z<z_{c}$, by the distributions of interevent times and ages, respectively. The second class includes HPTs of a MGCC in interdependent networks as well as HPTs of a giant *k*-core. As $z\to z_{c}^{+}$, the mean avalanche size of link failures diverges, producing a critical avalanche size distribution at $z_{c}$. In both classes, the mechanism underlying the critical avalanches at $z_{c}$ can be consistently interpreted within a unified framework as a critical branching process described by the SWIR model.

Strikingly, for both categories of HPTs, two separate families of critical exponents have been identified: one associated with the order parameter and the other with the corresponding size distributions. This broadened framework captures not only the standard scaling relations but also reveals additional critical features, including the coupled scaling relation connecting these two families, as expressed in Equation (11). We expect that this recent progress on HPTs will provide a basis for understanding a wide variety of hybrid phase transitions, such as hybrid synchronization transitions driven by frequency-degree correlations [50] and hybrid phase transitions in thermal systems [51,52] and colloidal crystals [53]. Finally, these results can help provide an understanding of other types of percolation transitions, including the recently reported hybrid universality classes of cascades [54], higher-order percolation processes [55], and percolation in hypergraphs [56,57].

## Figures and Tables

**Table 1:** Critical exponents in the HR model measured in [33,36].

*g*	$\beta_{m}$	$\gamma_{m}$	${\bar{\nu }}_{m}$	$\tau_{s}$	$\sigma_{s}$	$\gamma_{s}$	${\bar{\nu }}_{s}$	$\zeta^{\prime }$
0.2	0.09±0.05	0.90±0.10	1.05±0.10	2.08±0.04	0.99±0.05	0.91±0.05	1.10±0.10	1.89±0.10
0.5	0.21±0.05	0.83±0.10	1.25±0.15	2.18±0.04	0.96±0.05	0.83±0.05	1.24±0.11	1.76±0.13
0.8	0.32±0.05	0.76±0.10	1.40±0.15	2.25±0.04	0.89±0.05	0.81±0.05	1.42±0.13	1.65±0.13

**Table 2:** Critical exponents in the BFW model measured in [36].

*h*	$\beta_{m}$	$\gamma_{m}$	${\bar{\nu }}_{m}$	$\tau_{s}$	$\sigma_{s}$	$\gamma_{s}$	${\bar{\nu }}_{s}$	$\zeta^{\prime }$
0.2	0.06±0.01	1.05±0.07	1.18±0.06	2.04±0.02	1.11±0.11	0.87±0.07	0.96±0.08	1.99±0.08
0.5	0.24±0.01	0.93±0.12	1.42±0.11	2.21±0.03	1.04±0.08	0.76±0.03	1.26±0.19	1.79±0.03
0.8	0.33±0.02	0.85±0.16	1.51±0.13	2.30±0.05	1.02±0.11	0.69±0.03	1.33±0.24	1.74±0.08

**Table 3:** Critical exponents in percolation of interdependent networks measured in [44].

	$\beta_{m}$	$\gamma_{m}$	${\bar{\nu }}_{m}$	$\tau_{a}$	$\sigma_{a}$	$\gamma_{a}$	${\bar{\nu }}_{a}$
ER	0.5±0.01	1.05±0.05	2.1±0.02	1.5±0.01	1.0±0.01	0.5±0.01	1.85±0.02
2D	0.53±0.02	1.35±0.10	2.2±0.20	1.59±0.02	0.70±0.05	0.5±0.05	2.1±0.2

**Table 4:** Critical exponents in *k*-core percolation of ER networks measured in [48].

$\beta_{m}$	$\gamma_{m}$	${\bar{\nu }}_{m}$	$\tau_{a}$	$\sigma_{a}$	$\gamma_{a}$	${\bar{\nu }}_{a}$
0.5±0.01	0.97±0.01	2.06±0.05	1.5±0.01	1.0±0.01	0.52±0.01	2.0±0.01

## Data Availability

No new data were created or analyzed in this study. Data sharing is not applicable to this article.

## Abbreviations

The following abbreviations are used in this manuscript:

CPT	Continuous percolation transition
DPT	Discontinuous percolation transition
HPT	Hybrid percolation transition
EP	Explosive percolation
HR	Half-restricted
BFW	Bohman-Frieze-Wormald
MGCC	Giant mutually connected component
SF	Scale-free
ER	Erdos-Rényi

## References

[1] Broadbent S.R., Hammersley J.M. (1957). Percolation processes. Cambridge Philos. Soc..

[2] Flory P.J. (1941). Molecular Size Distribution in Three Dimensional Polymers. I. Gelation. J. Am. Chem. Soc..

[3] Flory P.J. (1941). Molecular Size Distribution in Three Dimensional Polymers. II. Trifunctional Branching Units. J. Am. Chem. Soc..

[4] Flory P.J. (1941). Molecular Size Distribution in Three Dimensional Polymers. III. Tetrafunctional Branching Units. J. Am. Chem. Soc..

[5] Murray J.D. (2005). Mathematical Biology.

[6] McLachlan D.S., Blaszkiewiczm M., Newnham R.E. (1990). Electrical Resistivity of Composites. J. Am. Ceram. Soc..

[7] Last B.J., Thouless D.J. (1971). Percolation Theory and Electrical Conductivity. Phys. Rev. Lett..

[8] Albert R., Jeong H., Barabási A.L. (2000). Error and attack tolerance of complex networks. Nature.

[9] Morone F., Makse H.A. (2015). Influence maximization in complex networks through optimal percolation. Nature.

[10] Watts D.J. (2002). A simple model of global cascades on random networks. Proc. Natl. Acad. Sci. USA.

[11] Shao J., Havlin S., Stanley H.E. (2009). Dynamic Opinion Model and Invasion Percolation. Phys. Rev. Lett..

[12] Erdos P., Rényi A. (1960). On the evolution of random graphs. Publ. Math. Inst. Hung. Acad. Sci..

[13] Stauffer D., Aharony A. (1994). Introduction to Percolation Theory.

[14] Christensen K., Moloney N.R. (2005). Complexity and Criticality.

[15] Baxter G.J., Dorogovtsev S.N., Goltsev A.V., Mendes J.F.F. (2012). Avalanche Collapse of Interdependent Networks. Phys. Rev. Lett..

[16] Lee D., Choi W., Kertész J., Kahng B. (2017). Universal mechanism for hybrid percolation transitions. Sci. Rep..

[17] Rozenfeld H., Gallos L., Makse H. (2010). Explosive percolation in the human protein homology network. Eur. Phys. J. B.

[18] Ziff R.M. (2009). Explosive Growth in Biased Dynamic Percolation on Two-Dimensional Regular Lattice Networks. Phys. Rev. Lett..

[19] Radicchi F., Fortunato S. (2010). Explosive percolation: A numerical analysis. Phys. Rev. E.

[20] Fan J., Meng J., Liu Y., Saberi A.A., Kurths J., Nagler J. (2020). Universal gap scaling in percolation. Nat. Phys..

[21] Araújo N.A.M., Andrade J.S., Ziff R.M., Herrmann H.J. (2011). Tricritical Point in Explosive Percolation. Phys. Rev. Lett..

[22] Lee H.K., Kim B.J., Park H. (2011). Continuity of the explosive percolation transition. Phys. Rev. E.

[23] Choi W., Yook S.H., Kim Y. (2011). Explosive site percolation with a product rule. Phys. Rev. E.

[24] da Costa R.A., Dorogovtsev S.N., Goltsev A.V., Mendes J.F.F. (2010). Explosive Percolation Transition is Actually Continuous. Phys. Rev. Lett..

[25] Grassberger P., Christensen C., Bizhani G., Son S.W., Paczuski M. (2011). Explosive Percolation is Continuous, but with Unusual Finite Size Behavior. Phys. Rev. Lett..

[26] Cho Y.S., Kahng B. (2011). Suppression effect on explosive percolation. Phys. Rev. Lett..

[27] Riordan O., Warnke L. (2011). Explosive percolation is continuous. Science.

[28] Cho Y.S., Hwang S., Herrmann H.J., Kahng B. (2013). Avoiding a Spanning Cluster in Percolation Models. Science.

[29] Panagiotou K., Sphöel R., Steger A., Thomas H. (2011). Explosive Percolation in Erdos-Rényi-Like Random Graph Processes. Elec. Notes in Discret. Math..

[30] Araújo N.A.M., Herrmann H.J. (2010). Explosive Percolation via Control of the Largest Cluster. Phys. Rev. Lett..

[31] Schröder M., Araújo N.A.M., Sornette D., Nagler J. (2017). Controlling percolation with limited resources. Phys. Rev. E.

[32] Trevelyan A.J., Tsekenis G., Corwin E.I. (2018). Degree product rule tempers explosive percolation in the absence of global information. Phys. Rev. E.

[33] Cho Y.S., Lee J.S., Herrmann H.J., Kahng B. (2016). Hybrid Percolation Transition in Cluster Merging Processes: Continuously Varying Exponents. Phys. Rev. Lett..

[34] Bohman T., Frieze A., Wormald N.C. (2004). Avoidance of a giant component in half the edge set of a random graph. Random Struct. Algorithms.

[35] Park J., Yi S., Choi K., Lee D., Kahng B. (2019). Interevent time distribution, burst, and hybrid percolation transition. Chaos.

[36] Choi H., Cho Y.S., D’Souza R.M., Kertész J., Kahng B. (2024). Unified framework for hybrid percolation transitions based on microscopic dynamics. Chaos Solitons Fractals.

[37] Ziff R.M., Ernst M.H., Hendriks E.M. (1983). Kineticsofgelation and universality. J. Phys. A Math. Gen..

[38] Buldyrev S.V., Parshani R., Paul G., Stanley H.E., Havlin S. (2010). Catastrophic cascade of failures in interdependent networks. Nature.

[39] Gross B., Volotsenko I., Sallem Y., Yadid N., Bonamassa I., Havlin S., Frydman A. (2025). The random cascading origin of abrupt transitions in interdependent systems. Nat. Commun..

[40] Gross B., Havlin S. (2025). Physical Realizations of Interdependent Networks: Analogy to Percolation. Entropy.

[41] Son S.W., Bizhani G., Christensen C., Grassberger P., Paczuski M. (2012). Percolation theory on interdependent networks based on epidemic spreading. Europhys. Lett..

[42] Cellai D., Dorogovtsev S.N., Bianconi G. (2016). Message passing theory for percolation models on multiplex networks with link overlap. Phys. Rev. E.

[43] Bianconi G. (2018). Multilayer Networks: Structure and Function.

[44] Lee D., Choi S.M., Stippinger M., Kertész J., Kahng B. (2016). Hybrid phase transition into an absorbing state: Percolation and avalanches. Phys. Rev. E.

[45] Chalupa J., Leath P.L., Reich G.R. (1979). Bootstrap percolation on a Bethe lattice. J. Phys. C.

[46] Dorogovtsev S.N., Goltsev A.V., Mendes J.F.F. (2006). *k*-Core Organization of Complex Networks. Phys. Rev. Lett..

[47] Baxter G.J., Dorogovtsev S.N., Lee K.E., Mendes J.F.F., Goltsev A.V. (2015). Critical Dynamics of the *k*-Core Pruning Process. Phys. Rev. X.

[48] Lee D., Jo M., Kahng B. (2016). Critical behavior of *k*-core percolation: Numerical studies. Phys. Rev. E.

[49] Janssen H.K., Müller M., Stenull O. (2004). Generalized epidemic process and tricritical dynamic percolation. Phys. Rev. E.

[50] Gómez-Gardeñes J., Gómez S., Arenas A., Moreno Y. (2011). Explosive Synchronization Transitions in Scale-Free Networks. Phys. Rev. Lett..

[51] Bar A., Mukamel D. (2014). Mixed-Order Phase Transition in a One-Dimensional Model. Phys. Rev. Lett..

[52] Jang S., Lee J.S., Hwang S., Kahng B. (2015). Ashkin-Teller model and diverse opinion phase transitions on multiplex networks. Phys. Rev. E.

[53] Alert R., Tierno P., Casademunt J. (2017). Mixed-order phase transition in a colloidal crystal. Proc. Natl. Acad. Sci. USA.

[54] Bonamassa I., Gross B., Kertész J., Havlin S. (2025). Hybrid universality classes of systemic cascades. Nat. Commun..

[55] Sun H., Bianconi G. (2021). Higher-order percolation processes on multiplex hypergraphs. Phys. Rev. E.

[56] Bianconi G., Dorogovtsev S.N. (2024). Theory of percolation on hypergraphs. Phys. Rev. E.

[57] Bianconi G., Dorogovtsev S.N. (2024). Nature of hypergraph *k*-core percolation problems. Phys. Rev. E.

